# Please keep calm: investigating hippocampal function without stress

**DOI:** 10.3389/fnbeh.2014.00356

**Published:** 2014-10-13

**Authors:** Petra Henrich-Noack

**Affiliations:** Neurobiology Group, Institute of Medical Psychology, Otto-von-Guericke University MagdeburgMagdeburg, Germany

**Keywords:** spatial behavior, hippocampal lesion, rat models, stress induced artifacts, dorsal CA1, vascular dementia, micro infarct

Please keep calm: investigating hippocampal function without stress

The article “Topographical disorientation after ischemic mini infarct in the dorsal hippocampus: whispers in silence” by Faraji et al. published in “Frontiers in Behavioral Neuroscience” is remarkable for three reasons:

- The behavioral “Ziggurat Test” used in this work is one of the most sensitive assessments available for detecting deficits in hippocampal function [because behavioral effects of such minor hippocampal lesions could not be detected so far (Driscoll et al., 2008)].
- Artifacts by stress or fear are avoided (as no aversive stimuli is used during the operant behavior).
- In conjunction with the model of micro brain lesions the depth of understanding brain function is surpassing (as the test goes far beyond a simple binary evaluation (function/no function) toward a multi-parameter assessment of the quality of behavioral changes).

Scientific methods are often used just as tools to quantify a chosen parameter. The aim is to apply a reliable test which gives a clear-cut result to proof a hypothesis. However, the interpretation of the numbers usually is not clear-cut: one should always have in mind that it is a substantial simplification to use scientific methods as mere tools: they are inevitably the subject of the research, too.

This issue is especially important when performing behavioral studies. The Morris Water Maze test (MWMT) since its publication (Morris, 1981) has been extremely helpful to investigate changes/deficits in spatial navigation in rodents and it has become a standard for such spatial learning and memory research. However, as in any behavioral tests, there are confounding factors, and here especially the stress rodents experience when they are placed into the water need to be considered. Therefore, it is not possible to know for sure whether the effects detected with a paradigm involving stress can be attributed to the concept of hippocampus-dependent spatial navigation or emotional components. That stress or fear may lead to confusing results has been demonstrated before, e.g., when it was described that lesions of the dorsal hippocampus induced rather emotional dysregulation than deficits in a context-dependent memory task (Henrich-Noack et al., 2011). Unfortunately, such stress and fear confounders are a drawback for most spatial navigation tasks (MWMT, fear conditioning, elevated mazes) and an appropriate model to detect early signs of vascular dementia has been missing so far. This is also the case because some of the behavioral tests avoiding stress and fear (e.g., variants of the t-maze or y-maze) have a rather simple conceptual design with restricted variables to be measured.

In their very elegant study Faraji et al. addressed this problem by using a newly developed behavioral test where the animals are trying to find baits on top of 16 pyramids which are equally distributed in a chamber. Importantly, here the rodents do not experience emotional stress during the task like it is the case when e.g., swimming in a water tank (and mild food restriction necessary for the task has only a negligible impact; Belda et al., 2005).

Other established spatial learning tests using appetitive motivation (e.g., the hole board) may also have the potential to detect behavioral effects of micro brain lesions, but it seem they have not yet reached such high sensitivity (Gordan et al., 2012). However, there have been studies recently presenting other new ways/techniques to overcome the problems of the standard tests: in addition to the reports introducing and applying the Ziggurate Test (Faraji et al., 2008, 2010, 2011); Sauvage et al. (2008, 2010) for example described innovative behavioral set-ups where one main aspect is also to investigate spatial and object recognition in rodents without the drawback of strong emotional components.

Beer et al. (2014) from this group have published data indicating that the dorsal CA1 shows higher activation for (non-spatial) object recognition tasks than the ventral CA1. The current work from Farajii et al. suggests that micro lesions in the dorsal hippocampus affect the performance in the Ziggurat Test more than ventral lesions: the number of returns was significantly higher than in unlesioned controls or after damage in the ventral hippocampal CA1 region. If the object recognition is more affected in animals with dorsal lesions (as Beer et al., 2014 suggest) it can be speculated that this failure may play a role in the increase in corrections (returns) in the Ziggurat Test. According to this interpretation these animals—which have this dorsal-CA1-lesion-induced specific deficit of object recognition—change, as a consequence, their strategy (i.e., more returns) in order to rely predominantly on creating a new spatial relation for a new, promising route.

Establishing new behavioral tasks is labor intensive, time consuming and risky—but the brain can only be understood by analyzing its function. Therefore, it is definitely worth the effort—as shown by the Faraji et al. publication. In addition, such work about new behavioral paradigms does not only answer many questions, it provides ample food for thoughts, lively discussions, thrilling ideas and new hypotheses, too. For example:

- As sex differences influence reliance on environmental cues (Tropp and Markus, 2001; Faraji et al., 2010) it would be interesting to see how (proestrous) female rats behave with such micro lesions in the dorsal CA1.
- An adapted version of the Ziggurat Test may be very helpful to investigate (early) behavioral deficits in other models of vascular dementia or in transgenic Alzheimer mice (Schneider et al., 2014) without the need for aversive stimuli.

As a conclusion, keeping the rats calm may induce motivation, emotions and enthusiasm in neuroscientists.

## Conflict of interest statement

The author declares that the research was conducted in the absence of any commercial or financial relationships that could be construed as a potential conflict of interest.
